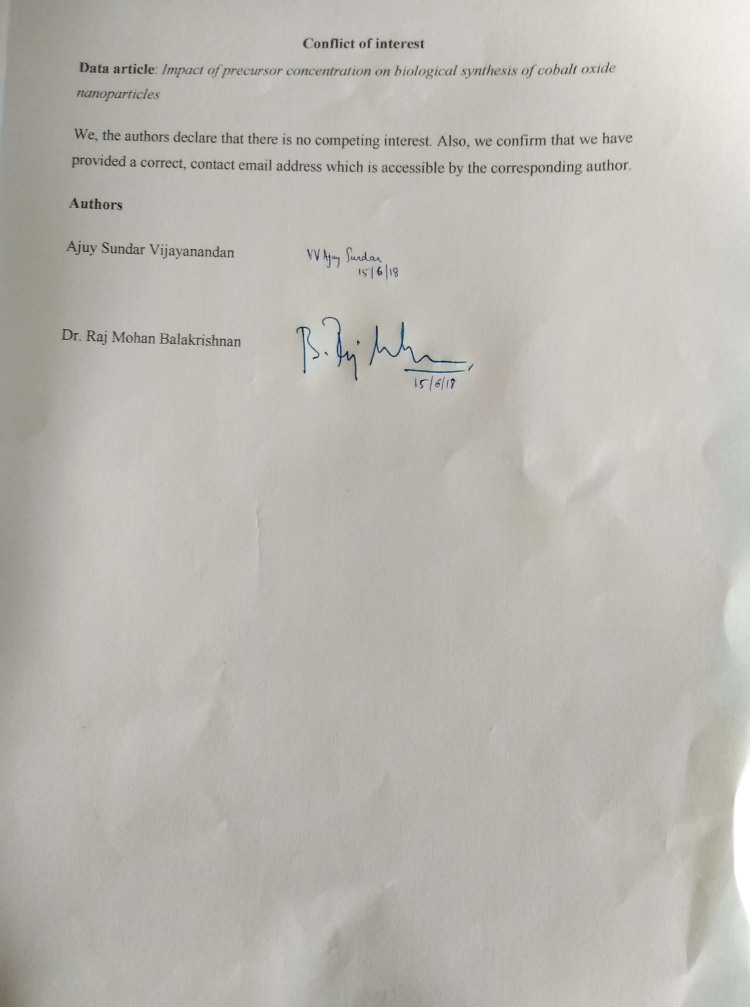# Impact of precursor concentration on biological synthesis of cobalt oxide nanoparticles

**DOI:** 10.1016/j.dib.2018.06.087

**Published:** 2018-06-30

**Authors:** Ajuy Sundar Vijayanandan, Raj Mohan Balakrishnan

**Affiliations:** Department of Chemical Engineering, National Institute of Technology Karnataka, Surathkal, Mangaluru 575025, India

## Abstract

The data provided in the article is in association with the journal article “Synthesis of cobalt oxide nanoparticles using endophytic fungus *Aspergillus nidulans*”. Characterization data (ultraviolet-visible (UV–vis) spectroscopy, dynamic light scattering (DLS) analysis, scanning electron microscopy (SEM), field emission gun scanning electron microscopy (FEGSEM)) of nanoparticles synthesized using different precursor concentrations (2 mM and 10 mM) have been presented in this article. Data obtained by *t*-test and *F*-test have been given for absorbance values exhibited by nanoparticles synthesized using different concentrations. Required figures and table have been depicted.

## Specifications Table

**Table:** 

Subject area	*Bionanotechnology*
More specific subject area	*Nanoparticle synthesis*
Type of data	*Figures (spectroscopy, microscopy), table*
How data was acquired	*UV–vis spectroscopy (Shimadzu UV-1800, Labomed USA), DLS analysis (Horiba Scientific Nanopartica SZ-100, Japan), SEM (JEOL-JSM-6380-LA, Japan)*, *FEGSEM (Field Emission Gun Nano Nova Scanning Electron Microscope 450, Thermofisher Scientific, USA), MaxStat Lite (MaxStat Software, Germany).*
Data format	*Filtered and analyzed.*
Experimental factors	*maximum absorbance peak (UV–vis), particle size distribution(DLS), morphology of particles (SEM, FEGSEM).*
Experimental features	*The solution containing nanoparticles was scanned from 200* *nm to 900* *nm spectral lines at room temperature in UV–vis analysis, DLS analysis was done with a scattering angle of 90° and medium viscosity of 0.89* *MPa s at 25* *°C, supernatant is taken directly for SEM and FEGSEM analysis*.
Data source location	*Department of Chemical Engineering, National Institute of Technology, Karnataka, Surathkal, Mangalore, Karnataka, India*
Data accessibility	*Data are with this article*
Related research article	*Title: Biosynthesis of cobalt oxide nanoparticles using endophytic fungus Aspergillus nidulans*[1]
	*doi:* 〈http://dx.doi.org/10.1016/j.jenvman.2018.04.032〉*.*

## Value of the data

• Different precursor concentrations of precursor salt cobalt acetylacetonate (2 mM and 10 mM) were used to synthesize cobalt oxide nanoparticles.
• UV–vis spectra, particle size distribution from DLS and SEM images of nanoparticles synthesized by 2 mM and 10 mM precursor concentrations were compared.
• *F*-test and *t*-test were performed to check the statistical significance of the absorbance data of nanoparticles obtained using 2 mM and 10 mM precursor salt concentrations.

## 1 Data

**Fig. 1:**
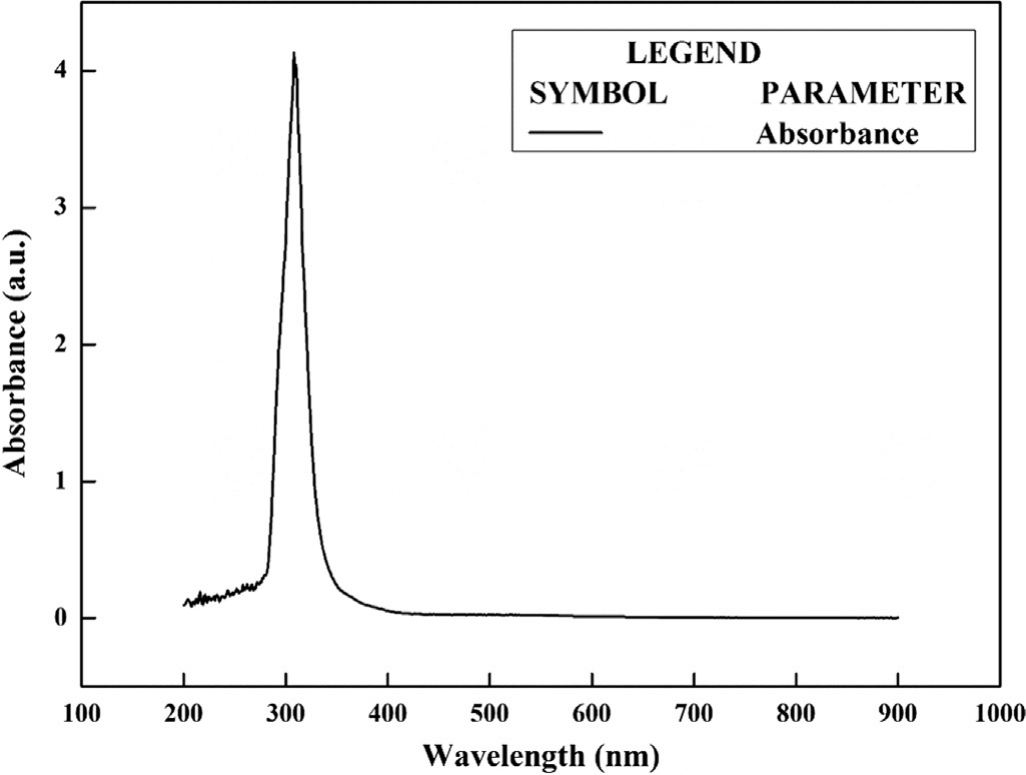
UV–vis spectra of nanoparticles synthesized using 2 mM precursor concentration.

**Fig. 2:**
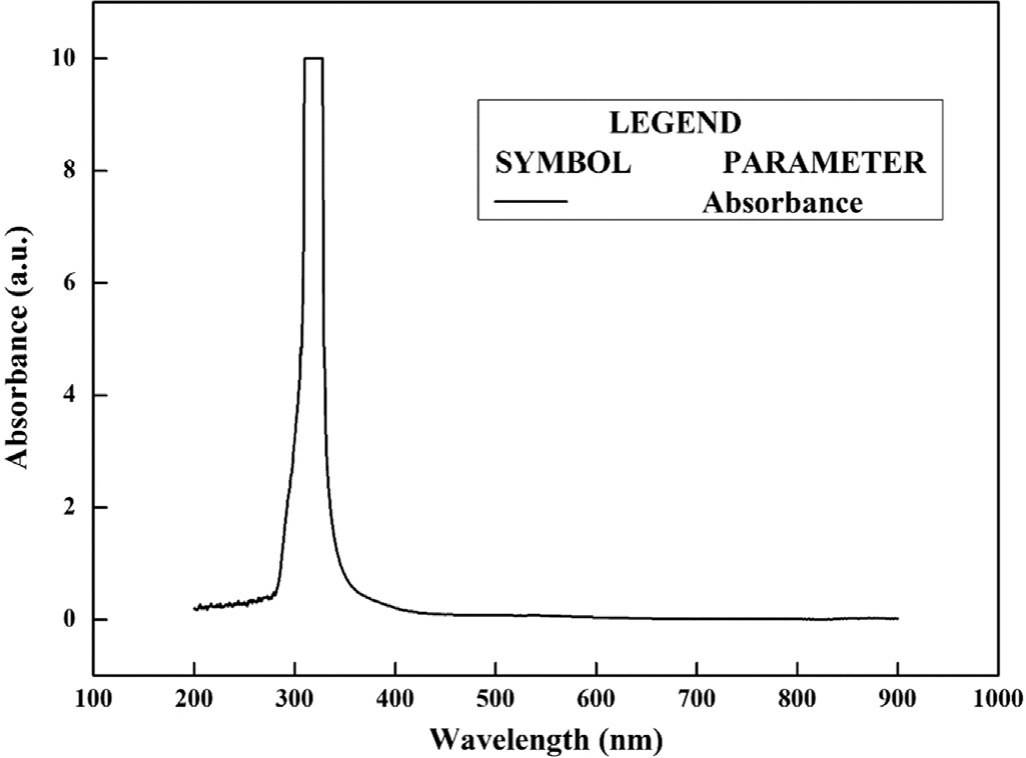
UV–vis spectrum of nanoparticles synthesized using 10 mM precursor concentration.

**Fig. 3:**
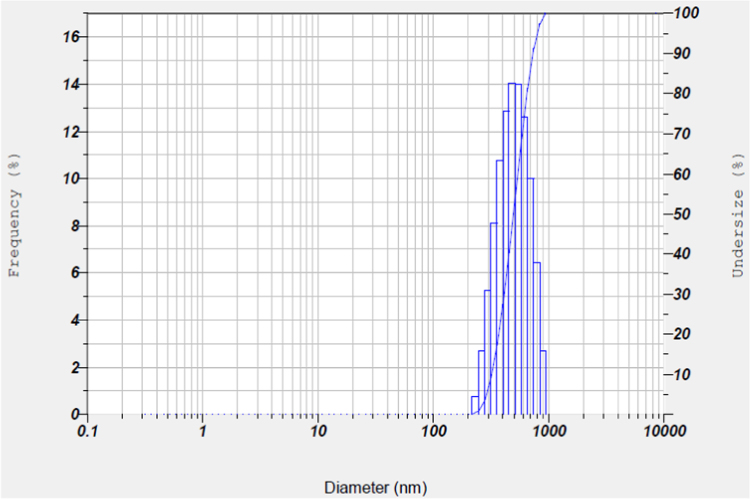
Particle size distribution of nanoparticles from DLS analysis (2 mM precursor concentration).

**Fig. 4:**
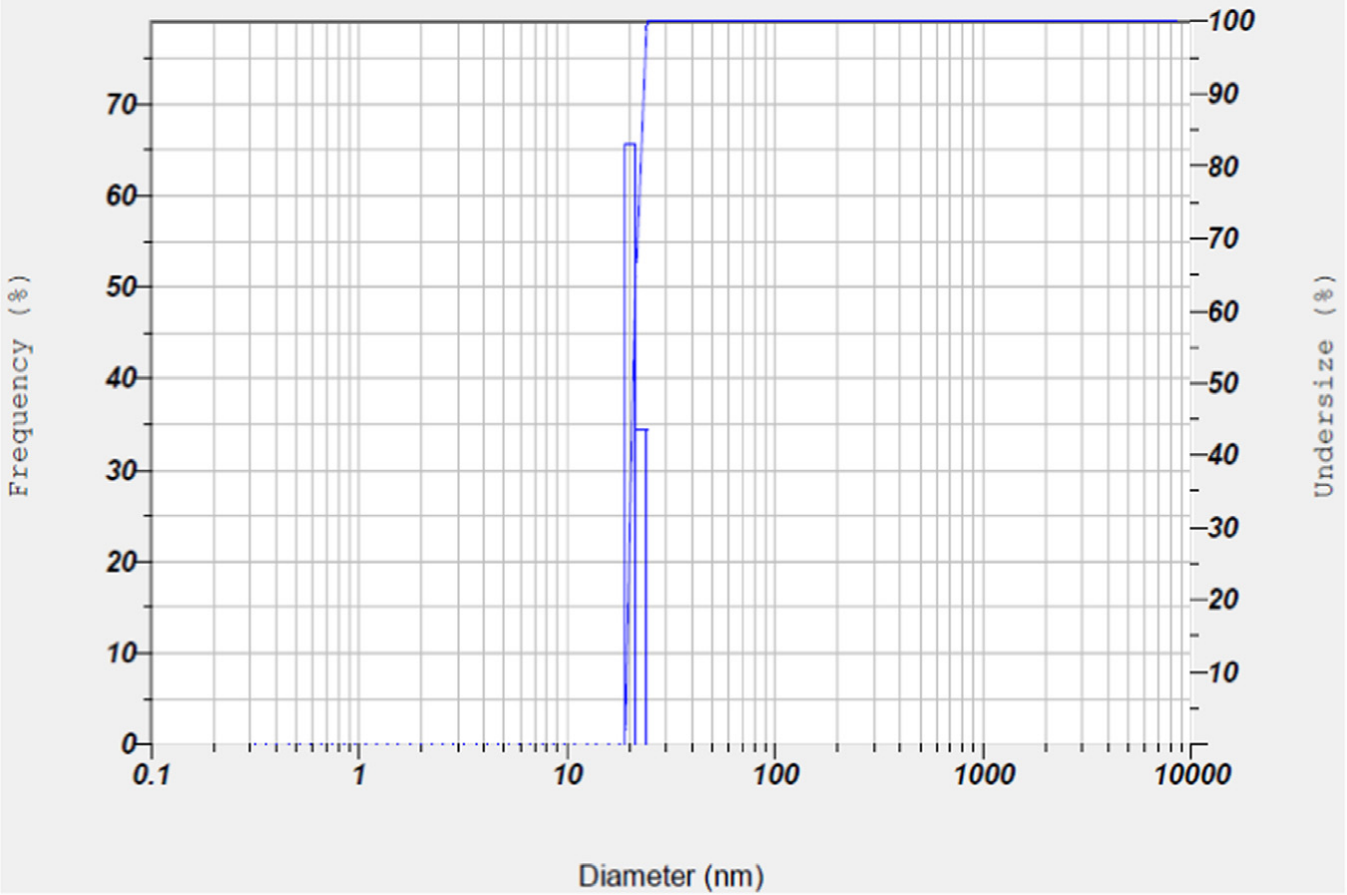
Particle size distribution of nanoparticles (10 mM precursor concentration).

**Fig. 5:**
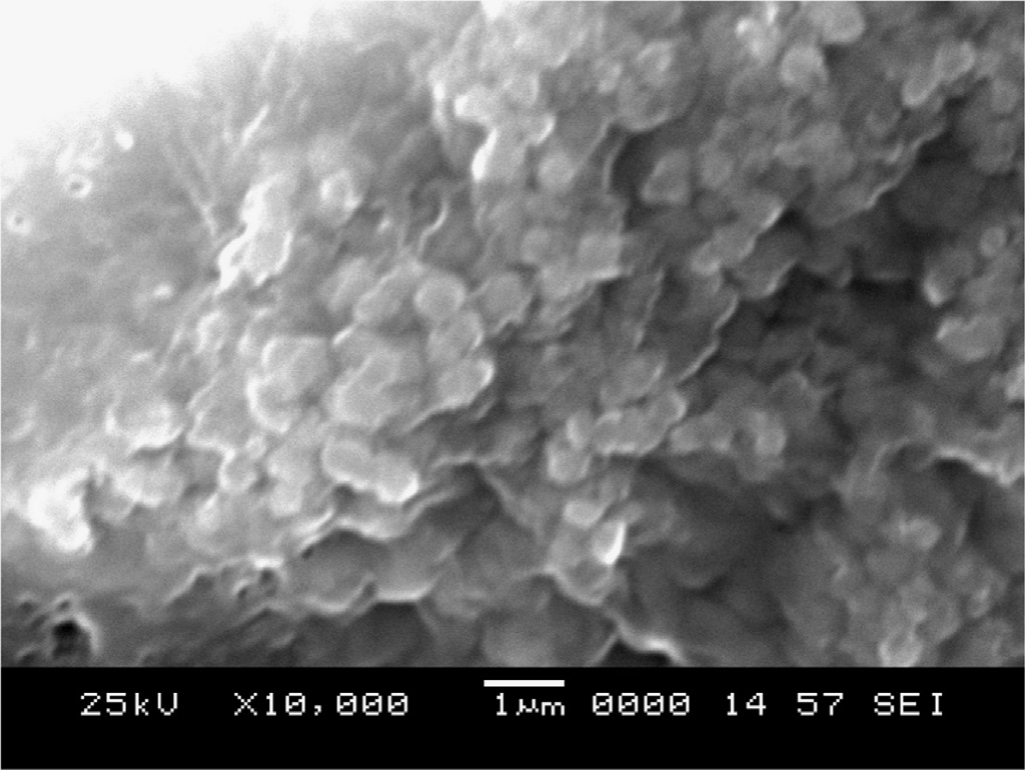
SEM image of nanoparticles (of 2 mM precursor concentration).

**Fig. 6:**
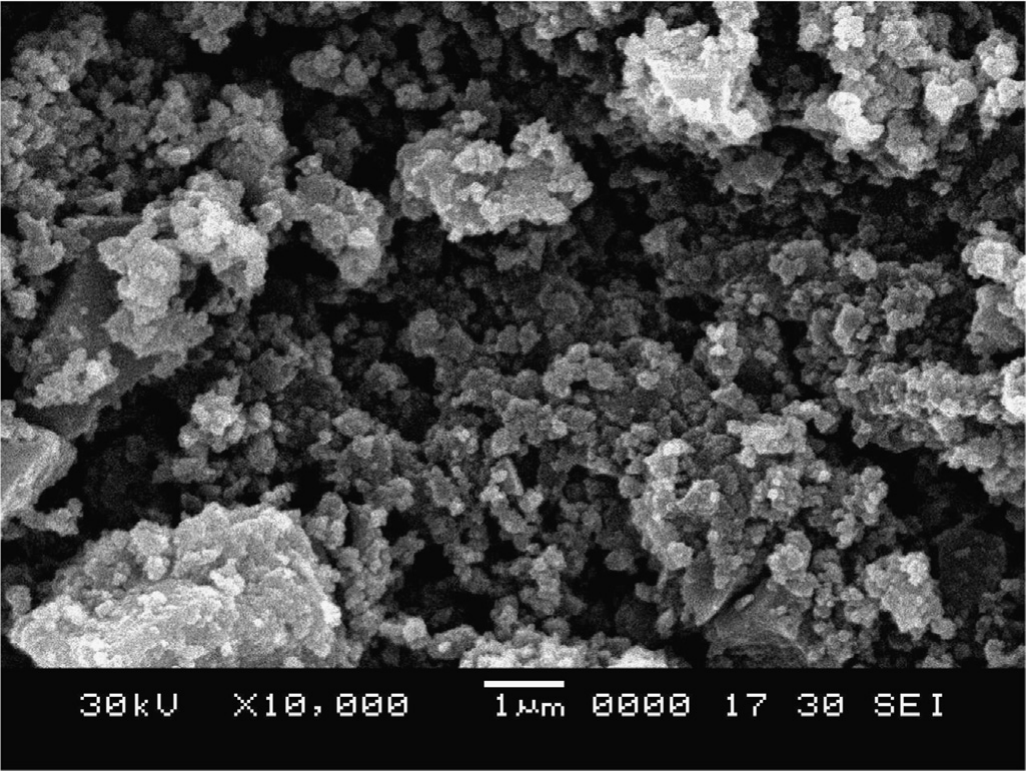
SEM image of nanoparticles (of 10 mM precursor concentration).

**Fig. 7:**
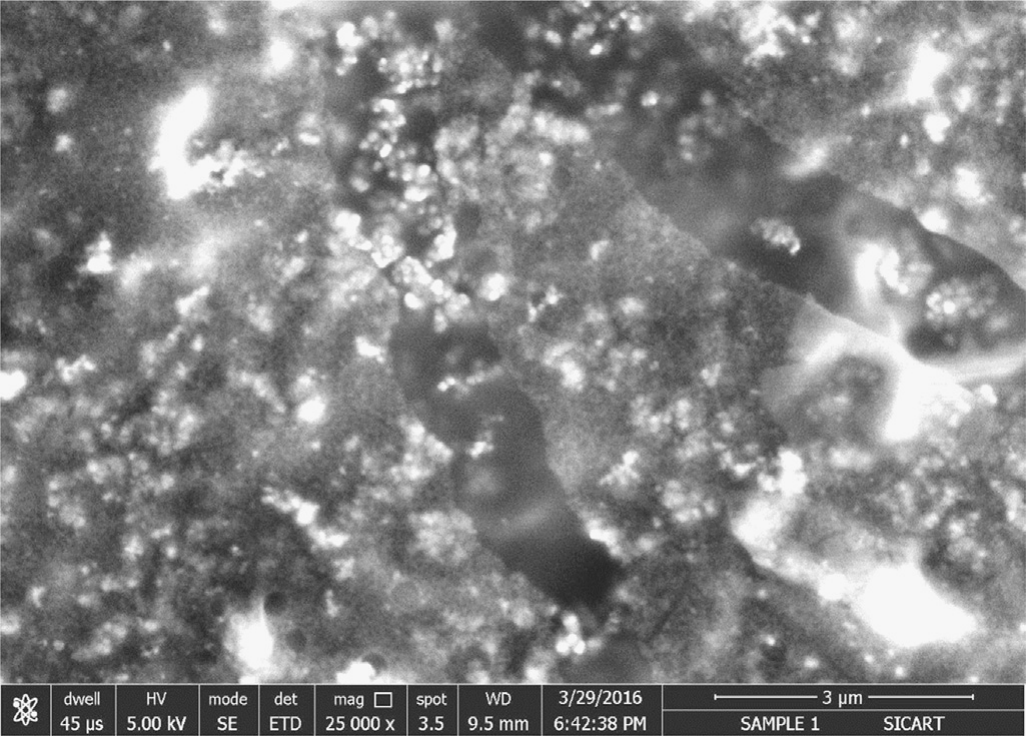
FEGSEM image of nanoparticles (of 2 mM precursor).

**Fig. 8:**
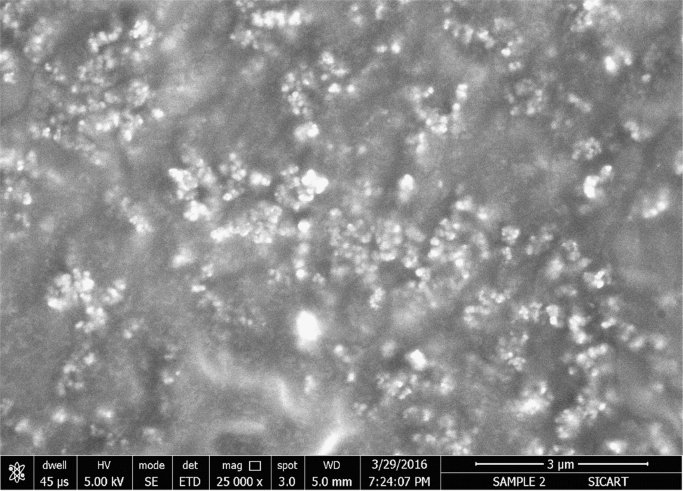
FEGSEM image of nanoparticles (of 10 mM precursor).

The synthesis of nanoparticles is done using fungal inoculum of *Aspergillus nidulans* and precursor salt concentration (cobalt acetylacetonate at 2 mM and 10 mM concentrations). The UV–vis spectrum of synthesized cobalt oxide nanoparticles using 2 mM precursor concentration is given in Fig. 1. Fig. 2 illustrates UV–vis spectrum of nanoparticles synthesized through 10 mM precursor concentration. Fig. 3 represents particle size distribution of nanoparticles (obtained from 2 mM precursor concentration) obtained from DLS analysis. Particle size distribution of nanoparticles (obtained from 10 mM precursor concentration) has been displayed in Fig. 4. Fig. 5, Fig. 6 show SEM image of nanoparticles synthesized by 2 mM and 10 mM precursor concentration respectively. FEGSEM images of nanoparticles synthesized have been displayed in Fig. 7, Fig. 8. Absorbance data obtained at 2 mM and 10 mM precursor concentrations from experimental work were statistically analyzed by t-test and F-test using MaxStat Lite (MaxStat Software, Germany), to determine the effect of precursor concentration on absorbance and statistical significance of data was determined using *p*-value and *F*-statistic (Table 1).

**Table 1:** Statistical analysis: *T*-test and *F*-test.

**Replicate**	**Absorbance at 2** **mM salt conc.**	**Absorbance at 10** **mM salt conc.**
1	1.193	1.711
2	1.192	1.690
3	1.172	1.689

***F**-***statistic**	***p*****-value**	**Are the means different? (*p* < 0.05)**
39	< 0.0001	Yes

## 2 Experimental design, materials, and methods

### 2.1 Materials

Potato dextrose broth (PDB) and chloramphenicol (Himedia Chemicals Pvt. Ltd., Mumbai), cobalt (II) acetylacetonate (Sigma Aldrich, Mumbai).

### 2.2 Synthesis

5 hoops of fungal inocula (*Aspergillus nidulans*) from the agar plate were taken and inoculated into a conical flask containing 100 mL of PDB medium. The flask was incubated in a rotary shaker at constant room temperature (30 ± 2 °C), 115 rpm for three days. The solution was filtered by Whatman paper No. 1 and biomass was rinsed three to four times with distilled water to remove the medium constituents. The washed biomass was transferred into 100 mL of 2 mM cobalt acetylacetonate solution and kept for stirring at the same condition mentioned above. After five days, the solution was filtered using Whatman paper No. 1 and supernatant was analyzed for the presence of nanoparticles [2], [3]. The precursor concentration was varied to 10 mM keeping the concentration of biomass, rpm and temperature same to study the effect of precursor concentration on the properties of nanoparticles.

### 2.3 Characterization

Cobalt oxide nanoparticles (synthesized using 2 mM and 10 mM precursor concentrations) were characterized by UV–vis spectroscopy and DLS analysis to determine maximum absorbance peak and particle size distribution respectively. The samples were morphologically analyzed using SEM and FEGSEM.

### 2.4 Statistical analysis

To determine the effect of precursor concentration on absorbance, absorbance data of nanoparticles collected using 2 mM and 10 mM precursor concentrations from experimental work were statistically analyzed by *t*-test and *F*-test using MaxStat Lite. The significance level (*α*) was taken as 0.05 and the statistical significance of difference between the means of absorbance was determined by comparing *p*-value with the value of significance, using null hypothesis. *F*-statistic was also estimated, which is the ratio of difference between sample means and difference within the samples.

## Figures and Tables

**Fig. S1:**